# An Integrated Treatment Approach for Bipolar II Disorder: A Clinical Case Study

**DOI:** 10.3390/jcm14238528

**Published:** 2025-12-01

**Authors:** Maria Theodoratou, Basant K. Puri

**Affiliations:** 1 Social Sciences School, Hellenic Open University, 263 31 Patras, Greece; 2 Department of Psychology, Health Sciences School, Neapolis University Pafos, 8042 Paphos, Cyprus; bpuri@cantab.net; 3 Cambridge Advanced Reseearch, Cambridge CB21, UK

**Keywords:** bipolar II disorder, hypomania, depression, integrated treatment, psychotherapy, lifestyle modifications

## Abstract

**Background/Objectives:** Bipolar II disorder is frequently misdiagnosed as unipolar depression, particularly when depressive symptoms predominate and hypomanic features are subtle or overlooked. This case study describes a patient initially treated for postpartum depression who later developed antidepressant-induced hypomanic symptoms, leading to the correct diagnosis of bipolar II disorder. The objective is to illustrate diagnostic complexities and highlight the value of an integrated treatment approach. **Methods:** Clinical assessment included standardized mood rating scales, structured interviews, functional evaluations, and monitoring of sleep and physical health indicators. Treatment combined mood-stabilizing pharmacotherapy with cognitive-behavioral therapy (CBT), dialectical behavior therapy (DBT), psychoeducation, and interpersonal and social rhythm therapy (IPSRT). Lifestyle interventions targeted sleep hygiene, physical activity, and stress management. **Results:** The diagnosis of bipolar II disorder was supported by the emergence of hypomanic symptoms following antidepressant treatment. The integrated therapeutic plan resulted in sustained mood stabilization, normalization of sleep patterns, improved occupational and social functioning, and reductions in depressive and hypomanic symptom scores. Physical health indicators, including body mass index, also improved. **Conclusions:** This case underscores the importance of comprehensive assessment and careful interpretation of antidepressant response in suspected bipolar presentations. A multimodal treatment approach integrating medication, psychotherapy, and lifestyle modification contributed to significant clinical improvement and may serve as a model for managing complex bipolar II presentations.

## 1. Introduction

Bipolar disorder represents one of the most challenging diagnostic and therapeutic frontiers in contemporary psychiatry, characterized by recurrent episodes of mania or hypomania alternating with depressive periods [1,2]. The condition affects approximately 1–2% of the global population and is associated with significant morbidity, functional impairment, and increased mortality risk [1,3]. Beyond the classic presentation described in diagnostic manuals, the clinical manifestation of bipolar disorder often includes subtle, frequently unrecognized features that complicate diagnosis and delay appropriate therapeutic intervention [3,4].

### 1.1. Diagnostic Challenges and Clinical Heterogeneity

A fundamental challenge in the diagnosis of bipolar disorder is distinguishing bipolar II disorder from recurrent unipolar depression, particularly when hypomanic episodes are brief, subsyndromal, or obscured by comorbid medical conditions [5,6]. The diagnostic criteria outlined in the Diagnostic and Statistical Manual of Mental Disorders (5th ed.) require a minimum duration and symptom threshold for hypomanic episodes; however, clinicians frequently encounter presentations that fall below these thresholds yet demonstrate clear treatment-response patterns consistent with bipolar illness [2,7]. This diagnostic ambiguity is particularly acute in primary care and non-specialist settings, where familiarity with the breadth of bipolar phenotypes may be limited and where delay to correct treatment is common [8,9].

Classical retrospective studies have identified predictive features of bipolarity such as earlier age of onset, frequent mood cycling, cyclothymic temperaments, and atypical antidepressant responses [5,10,11]. Family and pedigree studies further emphasize genetic loading and distinct familial patterns that increase diagnostic suspicion [12,13]. However, much of the literature is limited by retrospective designs, reliance on self-report or clinical interview that may miss brief hypomanic symptoms, and ascertainment bias from specialized psychiatric cohorts [3,7].

Diagnostic screening instruments can improve detection but are imperfect: brief patient-rated tools such as the Mood Disorder Questionnaire (MDQ) are helpful in many settings but may underdetect hypomanic or subthreshold states and require careful clinical follow-up [14,15]. Subthreshold hypomanic symptoms also contribute to progression from unipolar depression toward bipolar diagnoses in longitudinal studies, underscoring the need for longitudinal assessment and cautious interpretation of antidepressant response patterns [4,16].

### 1.2. Comorbidity and Diagnostic Complexity

Comorbid medical and psychiatric conditions greatly increase diagnostic complexity in bipolar disorder. Headache disorders, particularly migraine, are increasingly recognized in bipolar populations and may interact with illness course, treatment response, and quality of life [17,18]. The presence of migraine has been linked to greater illness complexity and, in some cohorts, to more rapid cycling, although prospective evidence clarifying causal directions is limited [17,18].

Beyond migraine, mood disorders in bipolar illness frequently co-occur with anxiety disorders, substance use disorders, attention-deficit/hyperactivity disorder (ADHD), and medical conditions such as asthma and metabolic disease, each of which contributes to diagnostic confusion and complicates treatment planning [2,19,20,21]. Network analyses and comorbidity research illustrate that symptom clusters (e.g., anxiety and mood instability) often interact dynamically and that comorbid conditions may escalate functional impairment and suicidal risk [22,23]. Clinical guidelines, however, provide relatively limited practical direction on optimizing pharmacotherapy when substantial somatic comorbidity is present [3,21].

Obsessive–compulsive symptoms and eating-disorder comorbidity can present episodic patterns in people with bipolar disorder, complicating differential diagnosis; these symptoms often intensify during depressive phases and remit during hypomanic periods, emphasizing the importance of time-linked symptom assessment [22,24]. Comorbid ADHD, particularly when present early in life, may mask or be misconstrued as mood instability, necessitating careful developmental history [25,26].

### 1.3. Clinical Case Report Literature: Contributions and Gaps

Case reports and small case series remain valuable for illustrating atypical presentations, medication-related destabilization, and decision points in diagnosis. They frequently provide granular timelines of symptom evolution, medication trials, and clinical reasoning that can illuminate diagnostic pitfalls encountered in routine practice [6,7,27]. Nevertheless, case literature often emphasizes treatment outcomes rather than diagnostic heuristics; comorbid medical conditions and the interpretation of subsyndromal hypomanic symptoms are underrepresented [7,28]. There is a need for case reports to include systematic diagnostic reasoning and to report dose-specific medication effects when relevant to differential diagnosis.

### 1.4. Novelty and Significance of This Case

This case study highlights several underrepresented diagnostic scenarios and clarifies how careful longitudinal assessment can refine diagnostic confidence: progressive identification of bipolar II disorder in a patient originally treated for unipolar depression, SSRI-related dose-dependent mood destabilization, and a notable interplay between migraine disorder and affective symptoms [7,17,27]. By documenting medication-specific responses, timing of somatic symptom emergence, and the patient’s longitudinal course, this report contributes to clinical guidance on recognizing complex and atypical bipolar presentations and the value of integrating psychosocial and somatic information when forming differential diagnoses [28,29].

### 1.5. Published Case Reports: Strengths and Weaknesses

Several published case reports have documented the diagnostic challenges associated with bipolar disorder. The atypical features—where manic symptoms resembled irritability rather than euphoria, while depressive episodes were more prevalent than manic episodes—are well-documented in elderly populations [30]. Medical comorbidities can indeed confound psychiatric diagnosis, as illustrated by complex cases involving dual systemic conditions [31]. Emphasis on non-traditional presentations represents a strength in case literature [32], although systematic clinical guidance for distinguishing bipolar disorder from mimics remains limited. The recognition that medical conditions can masquerade as or coexist with bipolar disorder underscores the importance of multidisciplinary assessment [33].

Existing case-report literature on bipolar disorder diagnostic challenges reveals several consistent strengths and limitations. Strengths include recognition of atypical presentations [30], documentation of comorbid medical and psychiatric conditions [31] and emphasis on longitudinal history assessment [34]. However, persistent weaknesses include insufficient attention to the role of antidepressants in precipitating or masking hypomania [35], limited discussion of mood stabilizer selection strategies in complex patients [33] and underrepresentation of cases where diagnostic clarity emerges only after treatment trials and careful observation. Moreover, few reports systematically address recognition of subsyndromal hypomanic features that precede full manic episodes [36]. Patient D’s case contributes by demonstrating how initial SSRI monotherapy can obscure the underlying bipolar diathesis [37] highlighting recognition of subtle hypomanic features [38] and integrating pharmacological management with acknowledgment of comorbid conditions [39].

## 2. Case Presentation and Clinical Context

### 2.1. Social History

D’s history illustrates the common delay in seeking mental health care, exemplified by chronic migraines and sleep disturbances dating back to college years [30]. Despite persistent symptoms, D did not see a mental health professional until adulthood, an oversight that can lead to delayed diagnosis and treatment of bipolar disorder [40]. This often results in antidepressants being prescribed without comprehensive mental health assessment [41]. Her upbringing in a middle-class, traditional family context involved considerable medical intervention for physical health problems, including recurrent bronchitis and whooping cough treated aggressively with antibiotics [33]. However, psychological well-being was consistently overlooked.

D’s childhood was marked by isolation and coping through literature [42]. The onset of severe asthma and worsening migraines coincided with her move to university, suggesting a link between emotional distress and psychosomatic symptom exacerbation [32]. The family’s tendency to overlook psychological factors, possibly due to societal stigma, created an environment where emotional health was consistently overshadowed [40]. Additionally, the patient has a family history of mood disorders, suggesting possible genetic predisposition [43].

### 2.2. Case Formulation

Patient D presented after experiencing a five-year history of mood disturbance, initially treated with sertraline 100 mg daily. After 18 months of SSRI treatment, she began experiencing markedly increased energy, decreased need for sleep (three to four hours nightly without fatigue), increased goal-directed activity, and racing thoughts—symptoms consistent with recurrent hypomanic and depressive episodes typical of bipolar disorder [44].

At age 35, D’s formal bipolar II disorder diagnosis occurred following postpartum depression [45]. Her first major depressive episode occurred approximately four months after childbirth, when she was initially treated with sertraline 100 mg/day [46]. Following SSRI initiation, she developed hypomanic symptoms including elevated mood, decreased need for sleep, and impulsivity—suggesting antidepressant-induced hypomania [37]. This temporal link between pharmacological treatment and hypomanic onset was central to the diagnostic process [47]. The recognition of her underlying psychological distress surfaced only after clear hypomanic breakthrough, revealing latent depression, heightened anxiety, and pressures from her responsibilities.

D’s early cognitive difficulties, first identified during primary school (around age eight), included deficits in attention, memory retention, and academic performance [48]. Following diagnostic criteria [2,48], D was diagnosed with bipolar II disorder, differentiated from major depressive disorder by the presence of hypomanic episodes [46].

D displayed hypersomnia, with sleep typically lasting 12–14 h [36] and her body mass index of 32.8 kg/m^2^ indicated obesity [39]. Her fatigue and oversleeping were initially attributed to the typical postpartum period [46]. However, her decision-making became increasingly erratic, and mental acuity and concentration diminished [43]. The emergence of a hypomanic phase, characterized by excessive talk, insomnia, and energy, coincided with irresponsible financial behavior, overcommitment to work, and difficulties with role functioning [49]. She exhibited symptoms of depression masked by physical symptoms, leading to prolonged depression and increased anxiety [39].

#### 2.2.1. Cognitive Deficits and Neuropsychological Profile

D’s early cognitive difficulties, first identified during primary school around age eight, included deficits in attention, memory retention, and academic performance [50], which are well-documented features of bipolar disorder [51]. These neurocognitive impairments represent persistent cognitive compromise that extends beyond mood episodes [52], suggesting a trait-like feature of her disorder rather than a purely state-dependent phenomenon [53].

Particularly notable is that her cognitive difficulties during depressive phases included problems with executive function and processing speed [54], domains consistently impaired in bipolar depression [55]. Her documented challenges with verbal recall and attention during the postpartum period align with research indicating that memory dysfunction particularly correlates with functional outcome in bipolar disorder [56]. The recognition that her depressive symptoms included both motor retardation and cognitive slowing [57] contributed to initial diagnostic confusion, as these features are sometimes misattributed to other medical conditions [58].

#### 2.2.2. Migraine as a Significant Comorbidity

Patient D’s chronic migraines dating back to college represent an important comorbid condition that warrants consideration. Migraine has been identified as a significant comorbidity in approximately 20–23% of patients with bipolar disorder [59,60]. The relationship between bipolar disorder and migraine comorbidity appears bidirectional, with increased prevalence of both conditions in each population compared to the general population [60].

Clinical research demonstrates that patients with comorbid bipolar disorder and migraine experience worse clinical outcomes, including more severe depressive symptoms [61], rapid cycling [62], and poorer quality of life [63]. The presence of migraine in D’s case, beginning in her college years and intensifying with the onset of mood dysregulation, supports the hypothesis of shared underlying neurobiological mechanisms [64]. Furthermore, comorbid migraine-bipolar disorder appears to constitute a distinct subphenotype requiring specialized treatment consideration [64].

#### 2.2.3. Obesity and Metabolic Dysfunction

D’s body mass index of 32.8 kg/m^2^ indicating obesity is clinically significant given the high prevalence of metabolic syndrome in bipolar disorder [65]. Research demonstrates that metabolic dysfunction in bipolar patients is associated with more severe cognitive impairment [66], particularly affecting executive function and processing speed [67]. Her obesity likely contributes to the neuropsychological deficits observed, as overweight and obese bipolar patients show significantly more pronounced brain imaging abnormalities than normal weight individuals [66].

The metabolic abnormalities in bipolar disorder result from multiple contributing factors, including medication-induced weight gain [67], lifestyle factors [68], and intrinsic illness factors [69]. In D’s case, the weight gain commenced following the initiation of psychiatric medications, particularly during the postpartum period when she began antipsychotic and mood stabilizer treatment [70]. The metabolic syndrome components—central obesity, dyslipidemia, and glucose dysregulation—are particularly concerning given evidence that they independently and synergistically increase cardiovascular mortality risk [71].

### 2.3. Diagnostic Distinction: Bipolar II Versus Unipolar Depression

The trajectory of D’s illness—characterized by depressive episodes punctuated by hypomanic breakthrough—is pathognomonic for bipolar II disorder [72]. Her initial five-year history of mood disturbance misattributed to unipolar depression represents a common diagnostic error [73], as many patients with bipolar disorder initially present with depressive symptomatology [74]. The critical diagnostic feature emerged only after SSRI initiation precipitated hypomanic symptoms [75], illustrating the diagnostic significance of antidepressant-induced mood switches.

Research demonstrates that approximately 6.5–21.6% of patients initially diagnosed with unipolar depression meet criteria for bipolar disorder [76,77]. Distinguishing features in D’s case include early age of symptom onset [78], multiple recurrent episodes [72], hypersomnia and weight gain during depressive phases [79], and family history of mood disorders [77]. The rapid cycling pattern she experienced—alternating between depressive and hypomanic episodes over months rather than years—is significantly more common in bipolar II than in unipolar depression [80].

### 2.4. Treatment-Emergent Complications and the Role of Antidepressants

The development of hypomanic symptoms following SSRI initiation (sertraline 100 mg/day) in the postpartum context represents antidepressant-induced treatment-emergent mania (TEM), a documented complication particularly relevant to bipolar disorder [81]. Meta-analytic evidence indicates that SSRIs carry significantly higher risk of mood destabilization in bipolar patients compared to other antidepressant classes [82]. The risk appears elevated in the immediate postpartum period when hormonal fluctuations and neurobiological vulnerability intersect [81].

Her case also illustrates why antidepressant monotherapy is contraindicated in bipolar depression [73]; SSRI use without concurrent mood stabilization substantially increases switching risk [81]. Current evidence-based practice now recommends that any patient presenting with depression should be screened for hypomanic or manic history before antidepressant initiation [71].

### 2.5. Differential Diagnosis

Accurate diagnosis is critical for optimizing patient outcomes, particularly in cases presenting with depressive symptoms [83]. Key differential diagnoses—including major depressive disorder, attention-deficit hyperactivity disorder (ADHD), cyclothymic disorder, borderline personality disorder, bipolar I disorder, schizoaffective disorder–bipolar type, substance/medication-induced mood disorder, and mood disorder due to a medical condition—were systematically considered and subsequently excluded based on clinical evaluation and history [84].

#### 2.5.1. Mood Stabilizers for Differential Diagnosis

Mood-stabilizing medications for bipolar disorder can be categorized according to their chronology of introduction and mechanism of action. First-generation mood stabilizers include lithium salts, which have proven efficacy in both manic and depressive episodes [85], as well as anticonvulsants such as valproate or divalproex sodium and carbamazepine, which demonstrate effectiveness in acute mania and maintenance treatment [86]. Second-generation mood stabilizers comprise atypical antipsychotics such as olanzapine, quetiapine, risperidone, aripiprazole, and lurasidone, which are increasingly utilized with evidence supporting their use in both acute and maintenance phases [87].

The selection of mood-stabilizing agents depends on several interconnected factors, including the polarity of predominant episodes (manic versus depressive), individual side-effect profile tolerability, relevant medical comorbidities, and prior treatment response patterns [88]. Lithium and valproate remain first-line agents with the most robust evidence base [89]. In patients presenting with bipolar depression complicated by migraine headaches, certain agents carry additional considerations; for example, valproate and some anticonvulsants demonstrate efficacy for both mood stabilization and migraine prevention, potentially addressing multiple symptom domains simultaneously [90,91].

#### 2.5.2. Distinguishing Bipolar II from Major Depressive Disorder

Distinguishing between bipolar II disorder and major depressive disorder is a critical component of psychiatric assessment [92,93]. Major depressive disorder is identified by isolated depressive episodes without the manic or hypomanic episodes characteristic of bipolar II disorder [2,94]. Preoccupation with major depressive symptoms can overshadow the critical need for mood-stabilizing therapies. The patient’s assessment showed hypomanic episodes, requiring the introduction of mood-stabilizing medications, such as lamotrigine, with consequent significant symptom improvement.

Early differential diagnosis between patients with major depressive disorder and bipolar disorder is essential for subsequently providing appropriate treatment [95,96,97,98]. Several validated assessment tools can facilitate this distinction [99]; affective temperament characteristics, particularly cyclothymic and anxious temperament scores assessed by questionnaires such as the TEMPS-A [100] and MDQ [14], can play supplementary roles in early identification.

#### 2.5.3. Diagnostic Challenges of Bipolar II and ADHD

ADHD manifests with symptoms similar to those of bipolar disorder, including attention problems, impulsivity, and organizational difficulties [101]. Adults with both ADHD and bipolar disorder tend to have earlier onset of bipolar symptoms, more frequent mood disturbances, and greater impulsivity [102]. However, important differences exist: in ADHD, symptoms of inattention and impulsivity persist across mood states, whereas in bipolar II disorder, these symptoms are typically restricted to manic or hypomanic episodes [103].

Features suggesting simultaneous diagnosis include earlier occurrence of bipolar-related symptoms (especially manic or hypomanic states), more severe course of the mood disorder, less adherence to treatment, and higher functional impact [91]. This comorbidity is associated with worsened symptom burden, increased psychiatric morbidity, and elevated suicide attempts [104,105]. A comprehensive clinical history with particular focus on neurodevelopmental history is important for accurate diagnosis of this comorbidity [106].

#### 2.5.4. Distinguishing Bipolar II from Cyclothymic Disorder

In cyclothymic disorder, symptoms fail to meet the symptom or duration criteria for hypomanic or major depressive episodes [107]. The patient’s episodes met full hypomanic criteria, excluding this diagnosis. Cyclothymic disorder is characterized by chronic mood instability with subthreshold mood episodes, whereas bipolar II disorder, by definition, requires at least one fully formed hypomanic episode.

#### 2.5.5. Distinguishing Bipolar I from Bipolar II

Bipolar I disorder is characterized by distinct manic episodes, whereas bipolar II involves at least one hypomanic episode [2,108]. The milder symptoms of bipolar II often lead to misinterpretation as mere mood swings. Despite inclusion as a distinct entity in the DSM since 1994, bipolar II disorder remains understudied and underrecognized, with patients typically experiencing symptoms for more than 10 years before receiving the correct diagnosis [109,110].

#### 2.5.6. Borderline Personality Disorder

While lability of mood and impulsivity occur in both bipolar II disorder and borderline personality disorder, key differences exist. In borderline personality disorder, mood shifts are typically rapid and occur in response to interpersonal stressors [111], whereas in bipolar II disorder, the mood changes are sustained and occur independent of external triggers [112]. In the present case, these symptoms represented distinct episodes with noticeable changes over baseline, favoring the diagnosis of bipolar II disorder.

The quality of mood fluctuations, impulsivity types, and linear progression of disorders should be carefully considered when differentiating the two [112,113,114]. Additionally, borderline personality disorder is more effectively managed with specialized forms of psychotherapy, while bipolar II disorder requires mood-stabilizing medications [113,114,115].

#### 2.5.7. Schizoaffective Disorder–Bipolar Type

This diagnosis was ruled out as the patient did not fulfill the criteria for concurrent psychotic and mood symptoms during periods of mood stability. Schizoaffective disorder requires the presence of a psychotic disorder as a core feature, distinct from bipolar II disorder where psychotic features, if present, occur only during mood episodes [116].

#### 2.5.8. Substance/Medication-Induced Mood Disorder and Mood Disorder Due to a Medical Condition

These were excluded as the patient had no medical history or relevant substance exposure deemed to be the cause of hypomanic episodes. A comprehensive medical and substance-use history is essential for ruling out these diagnoses, as medical conditions (particularly thyroid dysfunction, neurological disorders) and various medications or substances can precipitate mood symptoms that may mimic bipolar disorder.

### 2.6. Assessing Causation: Neurobiological and Psychosocial Factors

Research on the causation of bipolar depression has suggested a neurobiological basis [117,118,119,120]. While some studies have suggested a predominantly genetic basis for bipolar disorder, others have suggested a synergy of genetic, familial, and hereditary factors. Contemporary perspectives emphasize both neurobiological and psychosocial investigation

Immune dysregulation has received increasing attention as a central factor in the pathophysiology of bipolar disorder. Several aspects of immune dysregulations have been investigated, including low-grade inflammation with elevated cytokines such as IL-6 and TNF-α, as well as markers of peripheral immune activation. Understanding the distinct immune profiles characterizing bipolar versus unipolar depression is crucial for differential diagnosis and implementation of personalized treatment strategies [121,122,123,124,125].

The patient reported a family history of recurrent depressive disorder in a maternal aunt, supporting the possibility of a genetic predisposition. Family history of mood disorders in first-degree relatives is one of the most important risk factors for bipolar illness, particularly when multiple family members across generations are affected. This familial loading suggests involvement of genetic mechanisms in the etiology of the patient’s bipolar II disorder [126,127,128,129]. Following a thorough differential diagnostic evaluation, including assessment of mood fluctuation patterns, hypomanic episodes, and symptom course, the patient was diagnosed with bipolar II disorder. (Please see Table S1).

## 3. Therapeutic Interventions

### 3.1. Treatment Structure and Design

This single-case clinical illustration documents treatment outcomes for an adult patient with bipolar II disorder over a 12-month treatment episode, including a 6-month follow-up period. The study acknowledges the inherent methodological constraints of single-case design and does not claim to demonstrate efficacy or establish causal relationships [129]. The therapeutic approach was multimodal, guided by the need to address the patient’s affective instability, emotion dysregulation, and challenges with treatment adherence [130,131].

### 3.2. Assessment Rationale and Psychometric Protocol

The diagnostic and assessment protocol was informed by contemporary research emphasizing symptom interaction and psychometric sensitivity in affective disorders. A recent prospective 12-week treatment study of 143 patients from Villa dei Gerani in Catania, Italy [132], employed a dynamic network approach to identify the interaction of symptoms. This approach found utility in predicting the development of phenotype-related symptoms, thereby helping to inform the choice of therapeutic interventions [133]. Given the necessity of using a comprehensive psychometric protocol to account for the known variance in tool sensitivity to symptoms in Major Depressive Disorder and Bipolar II Disorder, assessment was conducted across multiple time points. The specific timelines and outcomes for both the pharmacological treatments and the integrated psychotherapeutic interventions are consequently presented for reference in Table 1.

### 3.3. Therapeutic Relationship and Commitment

Establishing a strong therapeutic relationship was considered the foundational context for the success of all subsequent psychotherapeutic modalities. This required the therapist to be versatile in their approach and highly skilled in managing the emotional peaks that can occur during therapy sessions [134,135]. Key skills employed included setting boundaries, accepting, empathizing, and understanding. It was imperative for the patient to feel safe and trusted to engage fully and articulate the anguish stemming from prolonged distress and repressed emotions. Crucially, the therapist’s task was to address her painful feelings without attributing blame for hereditary and biological predispositions, thereby significantly reducing her sense of guilt and self-criticism as she learned self-care strategies. Furthermore, encouraging the patient’s commitment to treatment, and involving family members when beneficial, was prioritized.

### 3.4. Pharmacological Interventions

The management integrated a multi-modal approach that combined necessary pharmacotherapy with structured psychotherapeutic modalities [136,137,138]. Pharmacological management adhered to contemporary guidelines [83] and included monthly psychiatric appointments. The pharmacological treatments used included lamotrigine as a mood stabilizer and sertraline, introduced cautiously for depressive symptoms [86,87,88,89,139,140]. The specific timeline of pharmacological treatments, including initiation dates and observed outcomes, is presented in Table 1.

### 3.5. Psychotherapeutic Interventions

The psychotherapeutic interventions implemented in this case are summarized in Table 2. These interventions were as follows: The psychotherapeutic approach fostered a multidimensional strategy to enhance emotional awareness, coping capacity, and functional stability [137,138]. The specific interventions are summarized in Table 2 and detailed below.

#### 3.5.1. Clinical Modalities

- Psychoeducation (Months 1–2, 6 sessions): Focused on bipolar disorder pathophysiology, medication adherence, and relapse prevention [141,142].
- Cognitive-Behavioral Therapy (CBT) (Weekly, 6 months): Implemented to identify and restructure maladaptive cognitions, challenge depressive thought patterns, and promote adaptive coping [143,144,145].
- Dialectical Behavior Therapy (DBT) (Weekly, Months 3–8): Addressed emotion regulation deficits and impulsivity using mindfulness and distress tolerance skills [146,147,148,149].
- Interpersonal and Social Rhythm Therapy (IPSRT) (Bi-weekly, Months 5–8): Targeted disruptions in daily structure and circadian rhythm irregularities [150].

#### 3.5.2. Behavioral and Lifestyle Modifications

Behavioral interventions were implemented to improve daily functioning and support the biological aims of IPSRT. These techniques are particularly valuable for individuals with bipolar disorder [151], especially in addressing sleep disruptions, which commonly arise in depressive and psychotic disorders and can lead to insomnia or hypersomnia [150]. Improvements in sleep quality are closely linked to enhanced mental health outcomes [152].

Sleep regulation strategies included elements of cognitive-behavioral therapy for insomnia (CBT-i) and the use of rapid awakening, both of which are commonly incorporated into psychotherapeutic treatment plans for sleep disorders and emphasize prompt transition from sleep to wakefulness with a short stabilization period for circadian adjustment [153,154,155,156,157]. Danilenko and Ivanova (2015) have further suggested that rapid awakening and dawn simulation may effectively reduce morning inertia and improve mood and daily functioning [158].

Lifestyle activation was another central component, with regular physical activity incorporated into the patient’s routine. Structured exercise programs have demonstrated significant benefits in improving mood and cognitive functioning, and their utility in reducing depressive symptoms in bipolar disorder has been extensively documented [159,160,161]. To further counter hypersomnia and excessive bed rest, the patient engaged in evidence-based strategies such as exposure to natural light, increased physical activity, social engagement, listening to uplifting music, and invigorating cold showers, which have shown effectiveness in managing these symptoms. A key psychological aspect of treatment involved developing personal boundaries [162,163] and self-management skills [164,165], as emotional volatility in bipolar disorder often complicates interpersonal stability. All interventions were selected collaboratively with the patient within a shared decision-making framework (please see Table S2).

## 4. Results

### Clinical Outcomes

The patient demonstrated clinical improvement across multiple domains, as quantitatively documented in the Comprehensive Outcomes Table (Table 3).

- Mood Stability and Episode Frequency: Initial assessment documented frequent mood oscillations (3 to 4 depressive episodes per year). Following 12 months of integrated treatment, the patient achieved stable mood with complete absence of major mood episodes. The Hamilton Depression Scale (HDRS) [166] score decreased from 28 at baseline to 12 at month 12, and the YMRS [168] score decreased from 12 to 3 (Table 3). Her follow-up MDQ score improved to 5/13.
- Sleep Pattern Normalization: Normalized sleep duration of 7 to 8 h was achieved by month 6 and sustained. PSQI [169] scores decreased from 14 at baseline to 6 at month 12 (Table 3).
- Physical Health Markers: Initial assessment revealed body mass index within the obese range. Following 6 months of treatment and lifestyle modifications, body mass index returned to normal range (25) and was maintained through follow-up (Table 3).

Suicide Risk Assessment: Suicidal ideation was assessed at each visit using the Columbia-Suicide Severity Rating Scale (C-SSRS); no suicidal ideation was reported.

Suicide risk assessment consistently remained negative (C-SSRS) throughout treatment (Table 3) [168].

- Medication Adherence: Initial assessment documented variable adherence. By month 3, consistent adherence to prescribed regimens was achieved and sustained [170].

Occupational and Interpersonal Functioning: Baseline occupational functioning was re-established by Month 9. Interpersonal relationships showed demonstrable improvement and a sustained reduction in conflict starting from Month 6. Acquisition of effective boundary setting and self-management skills proved critical to these gains. The patient (D) utilized psychoeducation to facilitate time management, and behavioral breaks were employed as a self-management strategy to regulate impulsivity or acute mood fluctuations. Stabilization of the patient’s social sphere, including distancing from manipulative individuals within complex, overly intertwined family dynamics, was essential for sustaining stability [159,160,171,172].

## 5. Discussion

### 5.1. Clinical Observations and Feasibility

This case illustrates the feasibility of implementing an integrated, multimodal treatment approach for bipolar II disorder. The observed clinical improvements across multiple domains—including mood stability, functional status, physical health, and medication adherence—demonstrate the value of the integrated approach. The synergistic combination of these approaches may have created additional benefits beyond those of individual components [166,167,168,169]. The case of D illustrates the effectiveness of a comprehensive approach to managing bipolar disorder. The patient’s recognition of her psychological and physical abilities was a breakthrough, which underscores the need for ongoing pharmacotherapy to influence her cognition, mood, and behavior.

### 5.2. Synergistic Effects

The integration of CBT (targeting depressive cognitions) may have been enhanced by concurrent DBT skills training (for emotional regulation) [143,144,145,146,147,148]. Similarly, IPSRT’s focus on social rhythm and relationships complemented the interpersonal skills developed through DBT, creating a comprehensive approach to social and temporal functioning [149].

A recent prospective study on major depressive and bipolar I disorder patients employed a dynamic network approach to identify the interaction of symptoms which helped inform the choice of therapeutic interventions [133,173]. Different psychometric tools vary in their sensitivity to detecting changes in cognitive and affective symptoms before and after treatment [173,174,175]. It has therefore been proposed that a comprehensive psychometric protocol be used to assess symptoms of bipolar disorders [176].

Establishing a strong therapeutic relationship was important, requiring skills such as setting boundaries, accepting, empathizing, and understanding [176]. The patient needed to feel safe and trusted to articulate the anguish stemming from prolonged distress and repressed emotions, as part of learning self-care strategies. The therapist’s task was to address her painful feelings without attributing blame for hereditary and biological predispositions, thereby significantly reducing her sense of guilt and self-criticism.

### 5.3. Limitations

This case study presents several limitations that should be considered when interpreting findings. First, the single-case design limits generalizability; results cannot be extrapolated to broader bipolar II populations without further investigation [177,178,179]. Second, the retrospective nature of some clinical data (particularly early childhood and adolescent symptoms) may introduce recall bias and limit diagnostic precision for earlier episodes. Third, without a control group, we cannot isolate the specific contribution of each therapeutic modality or determine whether improvements resulted from pharmacotherapy, psychotherapy, or their combination. Fourth, the extended timeline of treatment (from age 27 to 31) makes it difficult to attribute specific improvements to particular interventions, as multiple modalities were implemented sequentially and concurrently. Fifth, potential confounding variables—including family support, life changes, and the natural disease course—were not systematically controlled [180,181,182]. Finally, the findings reflect a single clinician–patient dyad and may not be replicable with different therapeutic relationships or treatment contexts. Despite these limitations, the case illustrates an integrated treatment approach that warrants investigation in larger, controlled studies.

## 6. Conclusions

The case of D illustrates the effectiveness of a comprehensive approach to managing bipolar disorder. By combining pharmacotherapy, psychotherapy, and lifestyle adjustments, D experienced significant improvements in symptom management and overall quality of life. The inclusion of CBT, DBT, and IPSRT reflects a tailored, evidence-based adaptation of standard bipolar disorder treatment frameworks. This multimodal, integrated model may serve as a paradigm for managing complex presentations following postpartum mood episodes. Effective management of bipolar disorder requires continuous, personalized care to meet the individual’s specific needs.

## Figures and Tables

**Table 1:** Timeline of Clinical Events/Symptoms, Assessment Findings, Interventions, and Outcomes.

Age (Years)	Clinical Event/Symptoms	Assessment Findings	Intervention	Outcome
Early childhood (6–10)	Recurrent bronchitis; emotional needs not always addressed	No psychiatric diagnosis at this stage	Antibiotic treatment for bronchitis	Physical health stabilized; emotional vulnerability noted retrospectively
Adolescence (15–18)	Academic underperformance; attention and memory difficulties	Early cognitive difficulties identified by teachers	Supportive educational strategies	Partial improvement; persistent difficulties
Early adulthood (23)	First hypomanic episode: elevated mood, reduced sleep, impulsive spending	DSM-5 criteria for hypomania met	No treatment initiated at this stage	Episode remitted spontaneously
Age 25	First major depressive episode: low mood, anhedonia, fatigue	HDRS (retrospective notes; moderate severity)	No structured treatment received	Functional impairment, withdrawal from studies
Age 27	Recurrent depressive episodes	Clinical evaluation; MDQ positive (10/13)	Initiation of pharmacological treatment: lamotrigine (100 mg/day)	Improved mood stability over 3 months
Age 28	Subthreshold hypomanic symptoms; interpersonal conflicts	YMRS mild elevation	Psychoeducation + CBT (focus on cognitive restructuring	Reduced impulsivity; improved insight
Age 29	Depressive relapse with rumination, sleep disturbance	HDRS moderate range	Addition of sertraline (50 mg/day) to lamotrigine	Gradual improvement; mild GI side effects resolved
Age 29–30	Emotional dysregulation; distress intolerance	DBT modules introduced (mindfulness, distress tolerance)	DBT skills training	Better emotional regulation; reduced interpersonal conflicts
Age 31 (current follow-up)	Stable mood; no suicidal ideation; functional recovery in work	MDQ 5/13; C-SSRS negative	Maintenance lamotrigine + psychotherapy (CBT + DBT)	Sustained remission of mood episodes

Scales that were used: HDRS, Hamilton Depression Rating Scale; MDQ, Mood Disorder Questionnaire; YMRS, Young Mania Rating Scale; C-SSRS, Columbia-Suicide Severity Rating Scale; CBT, Cognitive Behavioral Therapy; DBT, Dialectical Behavior Therapy; GI, gastrointestinal.

**Table 2:** Psychotherapeutic Interventions Summary.

Modality	Rationale	Core Components
**Cognitive-Behavioral Therapy (CBT)**	Targets depressive cognitions and promotes adaptive coping; evidence supports efficacy in bipolar disorder	Thought monitoring, cognitive restructuring, behavioral activation.
**Dialectical Behavior Therapy (DBT)**	Utilized to address emotion regulation deficits, impulsivity, and the impact of trauma/comorbid personality features	Mindfulness, distress tolerance, emotion regulation, interpersonal effectiveness.
**Psychoeducation**	Enhances medication adherence, self-monitoring, and recognition of prodromal symptoms; foundational for effective bipolar management	Illness awareness, relapse-prevention planning, communication skills.
**Interpersonal and Social Rhythm Therapy (IPSRT)**	Specifically designed for bipolar disorder to regulate the interplay between social stressors and underlying circadian rhythm disruption	Regular sleep–wake schedule, activity planning, interpersonal problem solving.
**Family Therapy**	Improves the support system and reduces expressed emotion (EE); complements individual therapy.	Communication skills, psychoeducation for family, relapse-prevention involvement.

**Table 3:** Comprehensive Clinical Outcomes.

Measure	Scale/Citation	Baseline	Month 6	Month 12	Month 18 (Final Follow-Up)
**Bipolar Screening**	MDQ (Hirschfeld et al., 2002) [14]	10/13 (Positive)	N/A	N/A	5/13 (Negative)
**Depression Severity**	HDRS (Hamilton, 1960) [166]	28 (Moderate)	16	8 (Remission)	10
**Mania Severity**	YMRS (Young et al., 1978) [167]	12 (Mild elevation)	6	3 (Normal)	4
**Suicide Risk**	C-SSRS (Posner et al., 2007) [168]	Not Reported	Negative	Negative	Negative
**Global Functioning**	GAF	55	68	78	76
**Functional Impairment**	FAST	32	20	12	14
**Sleep Quality**	PSQI [169]	14	9	6	7
**BMI (kg/m^2^)**	N/A	32.8 (Obese)	30.0	25.0 (Normal)	25.0
**Sleep Duration (Hours)**	N/A	12–14 (Hypersomnia)	7–8	7–8	7–8

## Data Availability

The data presented in this study are available on request from the corresponding author.

## Supplementary Materials

The following supporting information can be downloaded at: https://www.mdpi.com/article/10.3390/jcm14238528/s1, Table S1: Differential Diagnosis [condensed]. Table S2: Summary of Therapeutic Approaches in Bipolar II Disorder. Table S3: Table showing chronological sequence of psychological measures.
