# Supplementary material for: An Integrated Treatment Approach for Bipolar II Disorder: A Clinical Case Study
Source: J Clin Med. 2025 Dec 1;14(23):8528. doi: 10.3390/jcm14238528 (PMC12693252; doi:10.3390/jcm14238528)
Supplement: Supplementary file 1 [file jcm-14-08528-s001.zip › jcm-3933244-supplementary.pdf]

Table S1. Differential Diagnosis [condensed]

Diagnosis	Reason for Exclusion
Major Depressive Disorder	Absence of hypomanic episodes; MDQ > 10/13
ADHD	Symptom profile inconsistent; no pervasive inattentiveness across settings
Cyclothymic Disorder	Episodes met full hypomanic criteria, not subthreshold
Borderline Personality Disorder	Mood changes episodic, not pervasive; lack of identity diffusion
Bipolar I Disorder	No full manic episodes
Schizoaffective Disorder	No psychotic symptoms
Substance Induced Mood Disorder	No relevant substance exposure
Mood Disorder Due to Medical Condition	No medical condition causally linked to mood swings

Table S2. Summary of Therapeutic Approaches in Bipolar II Disorder

Therapeutic Modality	Primary Focus	Clinical Rationale
Psychoeducation	Understanding illness, adherence, relapse prevention	Improves insight and self-management; reduces stigma.
Cognitive-Behavioral Therapy (CBT)	Cognitive restructuring, behavioral activation	Addresses maladaptive beliefs and supports mood stabilization.
Dialectical Behavior Therapy (DBT)	Emotion regulation, distress tolerance, mindfulness	Enhances emotion control and treatment adherence; useful with affective instability.
Interpersonal and Social Rhythm Therapy (IPSRT)	Stabilizing daily and circadian rhythms	Targets sleep and activity regularity to prevent mood relapse.
Pharmacotherapy	Mood stabilization and symptom control	Lamotrigine and cautious SSRI use to balance depressive and hypomanic symptoms.

Table S3. Table showing chronological sequence of psychological measures

Time point	MADRS	HDRS	YMRS	MDQ (score / 13)	C-SSRS	GAF	FAST	PSQI
**Baseline** (pre-treatment)	28	Moderate severity (no	12	10	Negative (no suicidal	55	32	14

		numeric value reported)			ideation)			
**Month 6** (mid-treatment)	16	–	6	–	Negative	68	20	9
**Month 12** (end of active treatment)	8	–	3	5	Negative	78	12	6
**Month 18** (6-month post-treatment follow-up)	10	–	4	–	Negative	76	14	7
